# Toxin Degradation by Rumen Microorganisms: A Review

**DOI:** 10.3390/toxins12100664

**Published:** 2020-10-20

**Authors:** Zhi Hung Loh, Diane Ouwerkerk, Athol V. Klieve, Natasha L. Hungerford, Mary T. Fletcher

**Affiliations:** 1 Queensland Alliance for Agriculture and Food Innovation (QAAFI), The University of Queensland, Health and Food Sciences Precinct, Coopers Plains, QLD 4108, Australia; zhihung.loh@uq.edu.au (Z.H.L.); Diane.Ouwerkerk@daf.qld.gov.au (D.O.); AtholKlieve@outlook.com (A.V.K.); n.hungerford@uq.edu.au (N.L.H.); 2 Agri-Science Queensland, Department of Agriculture and Fisheries (QDAF), Ecosciences Precinct, Dutton Park, QLD 4102, Australia

**Keywords:** rumen microorganisms, plant toxins, probiotic, metabolism, degradation

## Abstract

Animal feeds may contain exogenous compounds that can induce toxicity when ruminants ingest them. These toxins are secondary metabolites originating from various sources including plants, bacteria, algae and fungi. Animal feed toxins are responsible for various animal poisonings which negatively impact the livestock industry. Poisoning is more frequently reported in newly exposed, naïve ruminants while ‘experienced’ ruminants are observed to better tolerate toxin-contaminated feed. Ruminants can possess detoxification ability through rumen microorganisms with the rumen microbiome able to adapt to utilise toxic secondary metabolites. The ability of rumen microorganisms to metabolise these toxins has been used as a basis for the development of preventative probiotics to confer resistance against the poisoning to naïve ruminants. In this review, detoxification of various toxins, which include plant toxins, cyanobacteria toxins and plant-associated fungal mycotoxins, by rumen microorganisms is discussed. The review will include clinical studies of the animal poisoning caused by these toxins, the toxin mechanism of action, toxin degradation by rumen microorganisms, reported and hypothesised detoxification mechanisms and identified toxin metabolites with their toxicity compared to their parent toxin. This review highlights the commercial potential of rumen inoculum derived probiotics as viable means of improving ruminant health and production.

## 1. Introduction

Common animal feeds including pasture grass, grains, crop residues, hay, silage and legumes or even water sources may contain toxins that can cause poisoning when animals ingest them. These toxins originate from secondary metabolites of plants, bacteria, algae or fungi and unlike primary metabolites are not directly part of the normal growth, development or reproduction of these organisms. Secondary metabolites by comparison are thought to protect against environmental stresses and predators, providing an advantage to the organism producing them [1]. Chemically, secondary metabolites comprise greater than 200,000 compounds which include compounds such as alkaloids, saponins, phenolic acids, steroids and terpenoids [2,3]. Once ingested, certain toxic secondary metabolites can interrupt the metabolic pathway of the animal consumer inducing undesirable biochemical and physiological changes in the cells, tissues and organs of the animal.

Toxic compounds can be commonly or seasonally found in ruminants’ feed or amongst pasture grass naturally where ruminants can inadvertently consume them thus inducing poisoning symptoms. Ruminants can develop adaptation mechanisms through rumen microorganisms to neutralise the effects of toxic secondary metabolites. These toxins are not toxic against rumen microorganisms and some can be used as energy sources for certain rumen microbial populations to adapt to toxins [4], with the detoxification capacity relying heavily on the amount of toxin consumed [5]. Rumen microbial populations are able to gradually change with prolonged, increasing exposure to toxins, thus allowing gradual tolerance of the toxin in ruminants [6]. Degradation pathways of a toxin in most cases involve a consortium of rumen microorganisms as the enzymes involved may not be present in a single rumen bacterial species [7]. In the case where a single rumen bacterium is capable of toxin degradation, there is a possibility of the presence of distinct microbial strains of the same species in the rumen that contributes to the detoxification process [8]. However, toxin degradation by rumen microorganisms does not always result in the detoxification of the toxins. For example, the mycotoxin zearalenone (ZEA) was reported to be degraded by rumen microorganisms but their metabolites were suggested to be more toxic compared to the parent toxin [9,10].

Various research studies have capitalised on the rumen’s capacity either through natural or augmented microbial abundance in the development of preventative probiotics. Probiotics are defined as live microorganisms which when administered in adequate amounts confer health benefits to the host [11]. Such probiotics are able to produce nutrients, produce or stimulate enzymes, provide competition with pathogens for adhesion sites, improve immune systems and metabolise or detoxify toxins [12]. An effective probiotic is generally characterised as a strain that can be beneficial to the host, has an appropriate count of viable cells, confers resistance to disease and toxins, yet is non-pathogenic, non-toxic and able to survive and metabolise in the gut environment [13]. Probiotics can be fed to naïve animals to confer resistance against the toxin thus allowing these animals to graze pasture grass or feed that contain toxins without severe toxic effects. These probiotics can be derived from either microorganisms in the environment which could degrade the toxins naturally or from rumen microorganisms of animals that are resistant to the toxin.

Our understanding of animal poisoning by toxic plants has grown rapidly over the last decade, along with new knowledge on the detoxification of plant toxins by adapted animals. However, most studies only report on the detoxification ability of studied microorganisms without describing the biochemical pathways for detoxification or their metabolites. This review mainly focuses on the detoxification of toxins found in plant or cyanobacteria and plant-associated fungal mycotoxins by rumen microorganisms and discusses the clinical studies of the toxicosis, toxin mechanism of action and biochemistry behind detoxification metabolism (Table 1). The review will include toxin degradation by rumen microorganisms producing reported and hypothesised less toxic or non-toxic metabolites (Table 2). It also includes both reported and hypothesised detoxification mechanisms of various toxins by rumen microorganisms, with particular emphasis on studies that have led or could potentially lead to the development of protective microbial interventions with the capacity to degrade the toxin for ruminants.

## 2. Rumen Microbial Detoxification of Plant Toxins

### 2.1. Non-Protein Amino Acids

Non-protein amino acids are plant secondary metabolites produced as a means to protect plants from environmental stresses, including herbivory [95]. Non-protein amino acids are structurally similar to proteinogenic amino acids which suggests both types of amino acids share similar synthetic metabolic pathways [96]. This would mean both types of amino acids compete for the same enzyme co-factors and transporters which can disrupt biochemical pathways [97]. Non-protein amino acids can be incorporated into protein chains leading to the formation of non-functional products that cannot be metabolised [98]. The presence of non-protein amino acids in fodder poses risks to livestock due to their potential toxicity and anti-nutritional properties [25,64,99]. There are *ca.*1000 known non-protein amino acids [98] but this review will focus on the small number of toxic amino acids currently known to be detoxified by rumen microorganisms.

#### 2.1.1. Mimosine

A well-recognised example of the development of a protective microbial consortium for detoxification of a plant toxin is the detoxification of the amino acid mimosine present in the leucaena plant (*Leucaena leucocephala*). Leucaena is a high-protein leguminous shrub, originating from the midlands of southern Mexico, Guatemala, El Salvador and Honduras [100], with use as a forage feed in tropical and subtropical agricultural systems starting in the 1950s in Australia [101] and Hawaii [102]. Feeding leucaena to cattle can result in leucaena toxicity with animals displaying symptoms including hair loss, goitre, inappetence and poor cattle live-weight gain [14]. Mimosine was reported to be degraded to 3,4-dihydroxypyridine (3,4-DHP) and 2,3-dihydroxypyridine (2,3-DHP) in the rumen [103], with these degradation products shown to be toxic [104].

A study reported that goats and cattle from Hawaii maintained appetite and exhibited no clinical signs of poisoning when fed leucaena exclusively [105]. By comparison, Australian ruminants exhibited marked hypothyroidism within four weeks [15]. An in vitro experiment, incubating leucaena in fresh rumen fluid showed Australian goat rumen fluid was able to rapidly convert mimosine to DHP but no further, whilst the Hawaiian goat rumen fluid showed a marked decrease in both mimosine and DHP levels [15]. A mixed bacterial culture, enriched from rumen fluid obtained from a Hawaiian goat, was then used to inoculate a steer in Australia which was fed leucaena for over 12 months without leucaena toxicity occurring and in vitro studies confirmed the presence of DHP-degrading bacteria [61]. Subsequently a novel anaerobic rumen bacterium, *Synergistes jonesii* was isolated with the ability to degrade 3,4-DHP to 2,3-DHP and 2,3-DHP to non-toxic products [62]. The development and production of a mixed anaerobic bacterial inoculum containing *S. jonesii*, produced in an in vitro fermenter, has enabled the adoption of leucaena as a fodder tree for cattle by Australian producers [63].

However, inoculation of buffalo rumen fluid containing *S. jonesii* into ruminants in eastern Indonesia did not fully confer mimosine resistance as there was no significant decrease in 2,3-DHP levels in the urine [106]. Halliday et al. [106] argued the possibility that the inoculum did not increase the presence of ‘functional’ *S. jonesii* in the rumen and was ineffective in conferring complete resistance to DHP toxicity. In China, four rumen-derived bacteria (two *Lactobacillus* spp., *Streptococcus bovis* and *Clostridium sporogenes*) were isolated and identified with the ability to substantially degrade mimosine, 3,4-DHP and 2,3-DHP under in vitro conditions [107]. A rumen bacterium that was able to degrade mimosine and DHP was isolated in Germany and identified to be an aero-tolerant gram-negative coccobacillus belonging to the genus *Klebsiella* [108].

**Figure 1:**
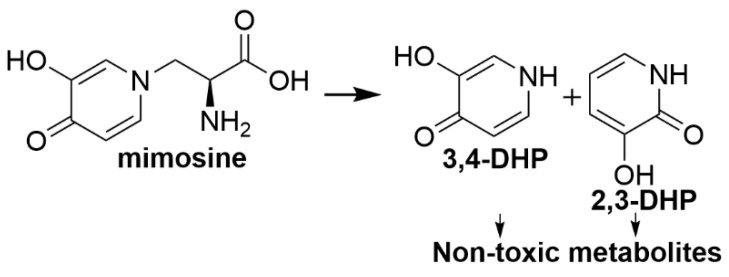
Proposed metabolite degradation pathway of mimosine (adapted from [111]).

**Figure 2:**
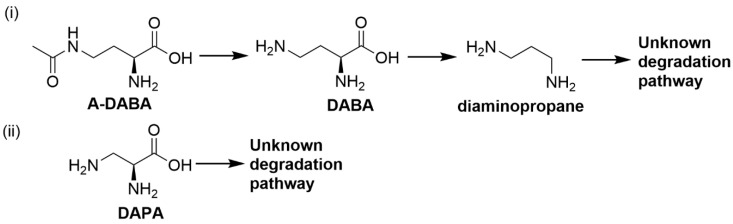
Proposed degradation pathway of (**i**) A-DABA and (**ii**) DAPA(adapted from [64]).

**Figure 3:**
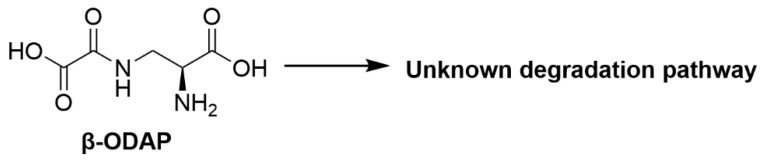
Chemical structure of β-ODAP.

Mimosine degradation (Figure 1) to 3,4-DHP involves the de-alkylation of the amine group, followed by isomerisation of 3,4-DHP to 2,3-DHP. Mimosine conversion to 3,4-DHP occurs via enzymatic reaction by endogenous rumen bacteria [103] and endogenous plant enzymes within leucaena leaves [104]. Isomerisation of 3,4-DHP to 2,3-DHP was suggested to be induced by an isomerase in *S. jonesii* whilst a dehydrogenase produced by *S. jonesii* was predicted to be responsible for the further degradation of 2,3-DHP [109]. Based on early studies, it was thought that mimosine detoxification would require synergism between a variety of rumen microorganisms, with Jones and Megarrity [15] demonstrating that there were a range of bacteria in the rumen able to degrade mimosine to 3,4-DHP. A number of bacteria have been isolated including *Streptococcus lutetiensis*, *Clostridium butyricum*, *Lactobacillus vitulinus* and *Butyrivibrio fibrisolvens* that are able to degrade mimosine to DHP [99]. Additional bacteria such as *S. jonesii* are required for the further breakdown of 3,4-DHP to 2,3-DHP which *S. jonesii* can further convert to unidentified non-toxic metabolites. Other rumen microorganisms may be also involved in the detoxification process in one way or another with roles ranging from stabilisation of the ruminal ecosystem to providing nutrients or co-factors required by the detoxifying bacteria [110].

#### 2.1.2. 4-*N*-Acetyl-2,4-diaminobuytric Acid (A-DABA)

*Acacia angustissima* is a tropical legume tree originating from Central America and has potential as a fodder tree for ruminants because of its high levels of protein. *A. angustissima* toxicity was first reported in sheep with poisoning symptoms including head pressing, grinding of teeth, foaming at the mouth and jerking of the body [16], although the toxin was not then identified.

Several secondary metabolites of *A. angustissima* were proposed to be toxic including condensed tannins, saponins and non-protein amino acids but their toxicity was not determined. Extracted condensed tannins of *A. angustissima* when fed to rats showed tannins contributed to the anti-nutritional effect but suggested the tannins were not the responsible toxins [112]. Non-protein amino acids such as 4-*N*-acetyl-2,4-diaminobutyric acid (A-DABA), diaminobutyric acid (DABA) and 2,3-diaminopropionic acid (DAPA) are commonly present in *A. angustissima* [113]. A feeding trial with naïve sheep showed adaptation when *A. angustissima* was introduced gradually, with increasing amounts fed over time resulting in no damage observed to their internal organs [16]. The study did not report on the presence of metabolites in the rumen nor isolate any toxin-degrading bacteria. Cross-inoculation of rumen contents from adapted sheep to non-adapted sheep conferred detoxification ability to the acceptor sheep even when fed high amounts of *A. angustissima* [16]. This result supported the hypothesis that rumen microorganisms play an important role in detoxification of toxins present in *A. angustissima* and these microorganisms can be transferred to non-adapted animals. A further study showed that DABA was the non-protein amino acid responsible for the toxicity in *A. angustissima,* as poisoning symptoms were observed to be similar to those caused by DABA in *Lathyrus sylvestris* (flatpea) [114].

A further in vitro study using enriched cultures obtained from rumen contents of sheep adapted to *A. angustissima* demonstrated degradation of A-DABA, DABA and DAPA [64]. A-DABA was observed to be hydrolysed to DABA (Figure 2i), as an intermediate, when incubated with the enriched culture in a medium without carbohydrates but no intermediate was observed in A-DABA incubated in media containing carbohydrates [64]. This suggests that the production of DABA as an intermediate of A-DABA degradation could be due to bacteria utilising A-DABA as a nutrient source in the absence of freely available carbohydrates. However, isolation of bacteria capable of degrading A-DABA and DABA was unsuccessful [64]. The presence of DABA aminotransferase and decarboxylase previously identified in several proteobacteria suggest the possibility that DABA is converted into diaminopropane in the rumen [64]. On the other hand, two rumen bacterial strains able to metabolise DAPA were successfully isolated and identified to belong to the *Firmicutes* phylum (strain LPLR3) and *Klebsiella* genus (strain LPSR1) respectively based on 16S rRNA gene analysis [64]. Strain LPSR1 showed degradation of DAPA in media containing carbohydrates while strain LPLR3 can grow and degrade DAPA in media containing DAPA as the main carbon source [64]. The authors hypothesised that DAPA degradation by strain LPLR3 was the result of its deamination to glutamate, as the molar amount of ammonia produced was equal to the amount of DAPA degraded (Figure 2ii) [64].

#### 2.1.3. β-*N*-Oxalyl-l-α,β-diaminopropionic Acid (β-ODAP)

*Lathyrus sativus* (grass pea) is widely cultivated in western Asia, North Africa and the Indian subcontinent as a forage crop with high dietary protein content [115]. The use of *L. sativus* however is limited due to the presence of β-*N*-oxalyl-l-α,β-diaminopropionic acid (β-ODAP) (Figure 3), a non-protein amino acid present in the seeds of *L. sativus*. Prolonged consumption of *L. sativus* seeds in humans is often associated with the development of lathyrism which is a neurodegenerative syndrome resulting in the paralysis of the lower limbs with β-ODAP identified as the toxin responsible [17]. Multiple studies were performed to elucidate the β-ODAP mode of action for inducing lathyrism but were unsuccessful [18]. β-ODAP was also proposed to induce oxidative stress and excitotoxicity which results in motor neuron degeneration [18,116]. Intraperitoneal administration of isolated β-ODAP to chicks at the level of 20 mg/chick resulted in the development of neurological symptoms and persisted up to 12 h while a higher administered dose of 30–60 mg/chick led to the symptoms persisting for up to 24 h and even resulted in death [17]. Pathological studies on the effects of β-ODAP in sheep showed nerve cell necrosis and degeneration in the cerebral cortex [117,118]. There are reported attempts to detoxify β-ODAP in *L. sativus* either in-field or post-harvest to achieve safe utilisation of *L. sativus* as food and fodder. *L. sativus* lines with low β-ODAP toxin levels were developed using traditional breeding methods but lines free of β-ODAP have not yet been successfully produced [119]. There are also efforts to genetically transform microorganisms with genes of enzymes capable of the hydrolysis of β-ODAP [120].

Efforts to isolate rumen microorganisms capable of degrading β-ODAP were reported. Enrichment and isolation of rumen bacteria able to degrade β-ODAP was carried out using rumen fluid of sheep (not previously exposed to *L. sativus*) incubated with culture medium containing concentrated β-ODAP extract as the sole source of carbon and nitrogen [65]. Three isolates were identified as able to degrade up to 46% β-ODAP in 24 h incubations [65]. The isolates were described morphologically to be cocci and to be different from each other but they were not identified [65]. Enrichment and isolation from cattle rumen contents incubated in medium containing β-ODAP as sole carbon source led to the isolation of six isolates capable of degrading β-ODAP, where five isolates were Gram-negative and one was Gram-positive [66]. 16S rRNA gene sequence analysis identified the isolates to be *Megasphaera elsdenii* with five different genotypes and *Clostridium bifermentans*. The isolates were also reported to degrade 96% of β-ODAP within six days of incubation [66], however the degradation product(s) was not reported.

#### 2.1.4. Indospicine

*Indigofera* spp. are leguminous herbs and shrubs with more than 700 species found mostly in subtropical and tropical regions [121]. These plants are of agronomical importance and widely used as grazing forages and feed supplements as they are rich in protein and palatable to livestock. However, the presence of indospicine in some *Indigofera* spp. has limited their agronomic potential. Indospicine (2(*S*)-2,7-diamino-7-iminoheptanoic acid) is a non-protein amino acid analogue of arginine which is present as the free amino acid in Indigofera plants [122]. Ingestion of indospicine was shown to cause severe liver damage to monogastric animals such as dogs, mice and rabbits [123,124,125]. Dogs are highly susceptible to indospicine hepatotoxicity with reported cases of secondary poisoning in dogs consuming indospicine-contaminated meat from horses and camels [126]. Indospicine could not be degraded in mammalian tissue due to the presence of the unusual amidino group as no known mammalian enzymes have the capacity to degrade this functional group [127].

Indospicine toxicity was observed in livestock consuming Indigofera plants with this amino acid confirmed to be the responsible toxin through in vivo rodent toxicity studies [124]. Further studies on indospicine described its toxicological effects in animals upon consumption. Since indospicine shares similar chemical structure to arginine, indospicine was hypothesised to be able to disrupt hepatic arginine-related protein synthesis leading to chronic liver damage. Indospicine was found to be a competitive inhibitor to rat hepatic arginase with arginase having similar affinity towards indospicine as to arginine, which negatively impacted aminoacylation of arginine causing urea cycle disruption [128]. Indospicine was also shown to interfere with hepatic protein metabolism resulting in fat accumulation and cytological changes in mice liver [124]. Indospicine was found to accumulate in animal tissues leading to secondary poisoning as indospicine can remain in tissues over a long period of time [123,129,130]. Indospicine has been shown to accumulate in cattle fed *I. spicata* with slow depletion rates (t_1/2_ more than 30 days in muscle) [122]. Indospicine is found to be a competitive inhibitor of arginyl-tRNA synthetase which prevents incorporation of arginine into tRNA affecting ribosomal polypeptide synthesis [128]. Purified indospicine was found to cause cleft palate in mice foetuses and had a high embryo-lethality [131].

Various studies reported ruminants grazing indospicine containing Indigofera plants, with different observations depending on the feeding dose and animal species. Cattle suffering from indospicine toxicity showed liver lesions, teratogenic and embryo-lethal effects [19]. There are reports of cattle able to tolerate indospicine under normal grazing circumstances in Australia, Hawaii and Kenya [132,133,134]. On the other hand, there are also studies which reported that cattle in Hawaii and Sri Lanka that were fed fresh Indigofera plants over a period of weeks showed clinical signs of poisoning with symptoms such as loss in body weight and liver lesions [21,134]. Cattle feeding on Indigofera plants were also reported to suffer from reproductive losses. Studies in Africa, Hawaii and Fiji showed that cattle that were fed with 16% to 100% Indigofera plants for their diet for over a month suffered abortions and stillbirths in pregnant cows and heifers [20,23,24]. These studies suggest that indospicine poisoning can be induced in cattle if they ingest large amount of Indigofera plants, with high intake of indospicine, over an extended period of time. In extensive pastures of northern Australia where cattle are not closely monitored, it is possible that similar instances of reproductive loss occur but are not readily attributed to protracted Indigofera consumption [19].

Sheep grazing Indigofera plants experienced both hepatotoxic effects and embryo-lethality [19]. Feeding trials in Hawaii showed that sheep on Indigofera diet displayed more clinical signs of poisoning compared to cattle, with sheep deaths occurring after 28 days. Post-mortem examination revealed fatty degeneration of the liver [134]. Sheep fed *I. spicata* in Australia developed signs of poisoning showing inappetence and weight loss with liver lesions [22]. Sheep are found to be able to tolerate small amounts of Indigofera without signs of poisoning, as also seen in cattle. Feeding trials with sheep consuming small amounts of Indigofera in Australia, India and Kenya reported no apparent signs of toxicity and Indigofera was highly palatable to sheep [132,133,135].

Indospicine which is an amidine was observed to be hydrolysed to the amide first, which is then further hydrolysed to the corresponding acid [67]. Amidine hydrolysis to amide is known to occur more rapidly under mild alkaline rather than acidic conditions [136]. Studies with pure indospicine in dilute aqueous sodium carbonate resulted in the hydrolysis of indospicine to 2-aminopimelamic acid, with further hydrolysis to 2-aminopimelic acid under dilute acidic conditions (1 N HCl, 120 °C, 2 h) [124]. Indospicine hydrolysis directly to 2-aminopimelic acid was also reported with concentrated hydrochloric acid (6 N HCl, 120 °C, 20 h) [124]. Indospicine was also found to be stable under mild acidic aqueous conditions in vitro and it was hypothesised that the mild acidic conditions in the stomach would not be able to hydrolyse indospicine [137]. 

**Figure 4:**
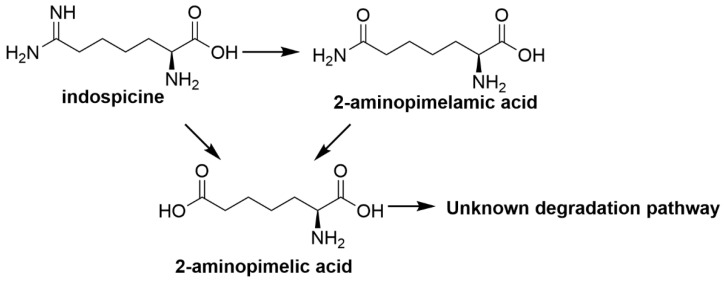
Hydrolysis pathway of indospicine (adapted from [67]).

Free amino acids are known to be taken up by rumen microbes in either native or deaminated form to produce ammonia as a nitrogen source for cellulolytic bacteria which means that indospicine could be metabolised via deamination pathways in the rumen [67]. Therefore, there is potential for rumen microorganisms to detoxify indospicine. Camel foregut fluid showed indospicine degradation of 99% in 48 h in an in vitro incubation study [67]. In a similar study, degradation of indospicine by cattle rumen fluid showed degradation levels of 97% after 48 h incubation [67]. However, the efficiency of indospicine degradation in vitro could not be translated to produce similar efficiency in vivo, as the solubility of indospicine allows the toxin to have a short ruminal retention time before moving into the intestine and being rapidly absorbed into blood plasma and tissues of animals. Cattle rumen metabolism of indospicine was hypothesised to be similar to that in camel foregut fluid. The camel is a unique non-ruminant herbivore that possesses a compartmental stomach and extensive foregut fermentation processes, such that researchers sometimes refer to camels as “pseudoruminants” [138]. The study also reported indospicine to be hydrolysed into 2-aminopimelamic acid and 2-aminopimelic acid (Figure 4) followed by further metabolism [67]. Although indospicine is an analogue of arginine, it does not follow a similar metabolic pathway to arginine when metabolised by rumen microbes. Instead, formation of 2-aminopimelamic acid was found to follow a similar pathway to the formation of citrulline from arginine, but the absence of nitrogen adjacent to the amide prevented ornithine equivalent metabolite formation [67]. There is no reported knowledge on the toxicity of both indospicine metabolites. The reported in vitro indospicine degradation by camel gut fluid and cattle rumen fluid suggested the potential of rumen microorganisms to be used as probiotics against the toxin. However, such ambition is perhaps compromised by the solubility of indospicine and the lack of binding of indospicine in tissues [123].

### 2.2. Fluoroacetate

Fluoroacetate (FCH_2_COO^−^) is a highly toxic compound, found in over 40 plant species [139], which is responsible for fatal poisonings of ruminants [25,26], and also used as a potent pesticide to control mammalian pest species [140]. The mechanism of fluoroacetate toxicity is well known, fluoroacetate is absorbed through the gut and converted to fluorocitrate, which binds strongly to the aconitase enzyme in the tricarboxylic acid (TCA) cycle thus terminating cellular respiration due to aconitase shortage [140,141]. Citrate builds up in tissues and blood, causing symptoms of toxicity including acidosis, hypocalcaemia and heart failure [27]. Fluoroacetate can be usually found in tropical and subtropical plants from the southern continents of Australia, South America and Africa belonging to the plant families of Fabaceae, Rubiaceae and Malpighiaceae [142], with such plants posing a significant risk to grazing livestock [19,78]. 

Despite studies reporting on fluoroacetate poisoning across many animals [143,144,145], some wildlife species are found to possess an innate tolerance to fluoroacetate compared to other animals. Animals in Australia such as *Tiliqua rugosa* (skink) were reported to have 100-fold tolerance to fluoroacetate [146] while *Dromaius novaehollandiae* (emu) that foraged in areas with fluoroacetate-accumulating plants were found to be 150 times more tolerant to fluoroacetate compared to emus foraging outside these areas [147]. Intraperitoneal administration of sodium fluoroacetate to tammar wallaby (*Macropus eugenii*) from Western Australia that was not exposed to any fluoroacetate-containing plants prior to the study showed tolerance against fluoroacetate poisoning [148]. These observations led to the proposed hypothesis that fluoroacetate tolerance is due to the lower metabolic rate in these animals which slowed the rate of fluoroacetate absorption and metabolism thus allowing these animals to excrete or detoxify fluoroacetate [149].

It was reported that feeding repeated low non-toxic doses of fluoroacetate containing plants to ruminants will confer resistance against fluoroacetate poisoning [150]. Furthermore, the resistance can be transferred to naïve animals through ruminal fluid transfer from experienced animals. Goats that were fed with increasing doses of fluoroacetate containing *Amorimia septentrionalis* in alternating periods, showed that the adapted goats possessed resistance against fluoroacetate poisoning while transfaunated goats, with adapted rumen fluid, showed clinical signs of poisoning much later compared to control goats [151]. Resistance against fluoroacetate poisoning was also observed in sheep fed with non-toxic doses of *Amorimia pubiflora* while transfaunated sheep, with adapted rumen fluid, showed initial clinical poisoning symptoms significantly later than the control group [150]. Although the above-mentioned studies did not report on any rumen bacteria isolations responsible for fluoroacetate degradation, it is predicted that these rumen bacteria would possess fluoroacetate dehalogenase activity capable of reductively breaking the carbon-fluorine bond in fluoroacetate [152]. Rumen microorganisms from un-adapted cattle rumen fluid that were enriched in the presence of fluoroacetate as carbon source resulted in the selection and isolation of a novel rumen bacterium capable of degrading fluoroacetate [68]. This bacterial strain was identified to be a *Synergistes* sp. and was able to degrade fluoroacetate (FCH_2_COO^−^) producing fluoride (F^−^) and acetate (CH_3_COO^−^) [68,69]. A recent study reported the isolation of rumen bacteria from the genera *Enterococcus* and *Bacillus* in media containing sodium fluoroacetate as the sole carbon source [153]. However, a study administering increasing subclinical doses of pure sodium fluoroacetate directly into a goat’s rumen did not result in the animal developing resistance against fluoroacetate [154].

An in vitro study using ovine rumen fluid, from an animal that did not previously graze fluoroacetate-containing plants, incubated in growth media with sodium fluoroacetate as the sole carbon source resulted in the isolation of two isolates capable of fluoroacetate degradation. 16S rRNA gene sequencing of both isolates identified as being *Pigmentiphaga* and *Ancylobacter* species [70]. Incubation of both isolates in media culture containing 20 mmol/L of sodium fluoroacetate resulted in the complete release of fluoride ions after 32 h of incubation [70]. A recent in vitro inoculation study using Australian cattle rumen fluid in culture medium containing fluoroacetate resulted in the isolation of a rumen bacterium which was identified as belonging to the *Synergistetes* phylum and in the genus *Pyramidobacter* [71]. The isolated *Pyramidobacter* strain was suggested to have similar fluoroacetate detoxifying functions to the previously reported bacterium from Davis et al. [68] and was found to be present in higher numbers, based on quantitative PCR analysis of collected rumen fluid, in cattle across northern Australia [71].

Experimental inoculation of ruminants with *Butyrivibrio fibrisolvens* bacteria genetically modified with a dehalogenase gene from the soil bacterium *Moraxella* sp. strain B showed success in preventing fluoroacetate poisoning in ruminants [72]. A further study involving genetically modified *B. fibriosolvens* strains transformed with dehalogenase genes inoculated into sheep showed the inoculated sheep to be resistant against fluoroacetate poisoning [73]. However, concerns over transgenic bacteria prevented the commercial use of such an approach to mitigate fluoroacetate poisoning in ruminants.

### 2.3. Pyrrolizidine Alkaloids

Alkaloids are a diverse group of amino acid-derived and nitrogen-bearing molecules present in plants. They are low molecular weight structures which make up 20% of plant based secondary metabolites. Alkaloids are known for their diverse bioactive properties ranging from toxicity to pharmacological properties [155,156]. Pyrrolizidine alkaloids (PA) are secondary metabolites produced by about 3% of all flowering plant species as protection against herbivory [157]. There are more than 660 PAs and *N*-oxide derivatives identified in over 6000 plants of four families, Apocynaceae, Asteraceae (Compositae), Boraginaceae and Fabaceae (Leguminosae) with half of these PAs reported to have toxic activities [158]. PAs are heterocyclic compounds consisting a necine base esterified with one or more necic acids. Generally, naturally occurring PAs present in plants are esterified necines and frequently occur as the *N*-oxide (PANO) [159]. PAs can be classified into four groups based on the structure of the necine base which are retronecine, heliotridine, otonecine and platynecine. Necic acids are aliphatic carboxylic acids derived from amino acids, ranging from simple acids to more complex di- and mono-carboxylic acids containing 7, 8 or 10 carbons, and are joined to the necine bases at either the 7-OH or 9-OH positions [159].

The toxicity of PAs is well documented. PA intoxication can cause acute, sub-acute or chronic toxicity where acute intoxication manifests as haemorrhagic necrosis, sub-acute toxicity causes blockage of hepatic veins, and chronic toxicity can lead to liver failure and death [159,160,161]. PAs of heliotridine-, retronecine- and otonecine-types are known to cause genotoxicity and tumorigenicity through formation of DNA adducts with dehydropyrrolizidine alkaloids (DHPA) [159]. PAs also undergo photosensitization causing oxidative stress and lipid peroxidation leading to tumour formation [162]. PAs are also reported to cause lung damage when DHPA travels into the pulmonary arterioles causing thrombi in vessels and thickening in lung walls leading to occlusion and inflammation [163]. Neurotoxicity by PAs is seen in necrotic lesions in the central nervous system [164].

**Figure 5:**
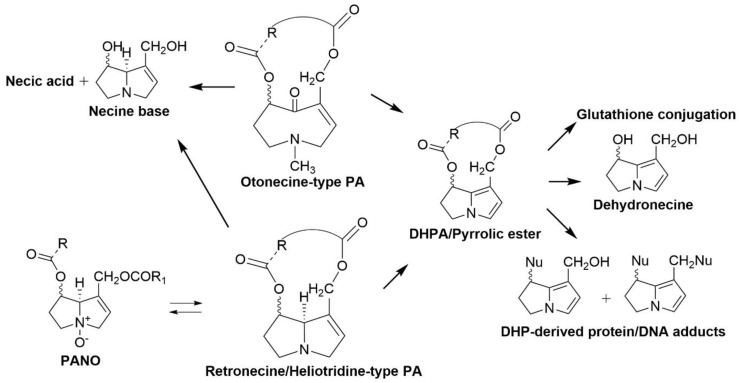
Retronecine/Heliotrine-type PA metabolism (adapted from [159]).

Metabolism of PAs is required for toxicity bioactivation. PAs are usually orally ingested and absorbed into the body from the gastrointestinal tract [159]. Bioactivation of PA mostly occurs in the liver thus making this organ the most affected by PA toxicity. In general, there are three principal PA metabolic pathways. The first metabolic pathway involves hydrolysis of the ester groups linked to the C_7_ and C_9_ positions to produce necines and necic acids by liver microsomal carboxylesterases [160]. The second metabolic pathway is the *N*-oxidation of the necine base of PAs forming pyrrolizidine alkaloid *N*-oxides which can be conjugated allowing for excretion [160]. The third metabolic pathway is the oxidation of PA to form pyrrolic esters or DHPA [160]. Hydrolysis and *N*-oxidation of PA are considered to be the main detoxification route for PA removal from the body [165]. However, PA *N*-oxides can be converted back into PAs and be oxidised into DHPA by CYP450 monooxygenases, notably CYP3A and CYP3B [163]. Not all PA types can be metabolised by all three pathways. Retronecine- and heliotridine-type PAs can be metabolised by all three principal metabolic pathways (Figure 5) [159]. On the other hand, otonecine-type PAs have necine bases which are structurally different than retronecine- and heliotridine-type PAs and oxidative *N*-demethylation of the necine base precedes further metabolism [159].

PAs are highly toxic to both monogastric and ruminant herbivores and present a constant issue for livestock industries [166]. Furthermore, the ability of PAs to be transferred and contaminate food products such as honey, milk and dairy products should be considered as a severe health risk [166]. Ruminants are resistant to PAs and are seldom reported to show acute poisoning but experience sub-acute to chronic toxicity [32]. Clinical symptoms of ruminant PA intoxication include loss of appetite, diarrhoea and depression [28]. Livestock losses were reported in the United States due to livestock grazing PA containing plants of *Senecio jacobaea* (tansy ragwort) [29] while PA intoxication of poultry, cattle, horses and pigs in Australia has identified PA containing plants *Heliotropium europaeum* (European heliotrope) and *Echium plantagineum* (Paterson’s curse) to be responsible [167,168,169,170,171]. Calves fed with *S. jacobaea* in doses of 1.3 kg/day (3 mg/kg bodyweight (bw) PA) resulted in megalocytosis after 182 days [32]. Calves fed with a single dose of 60 mg/kg bw PAs from *Cynoglossum officinale* (hound’s tongue) resulted in animal deaths within 48 h, whilst daily cattle feeding PAs with a dose of 15 mg/kg bw for 21 days showed hepatocellular necrosis by day 35 [30]. Goat kids and lactating dairy goats fed dried *S. jacobea* as 25% of a complete diet reported chronic clinical symptoms while cattle and horses with intakes of 0.05 and 0.20 kg/kg bw of *S. jacobea* respectively were lethal [31]. The study suggested that goats are more resistant to PAs compared to cattle and horses [31].

**Figure 6:**
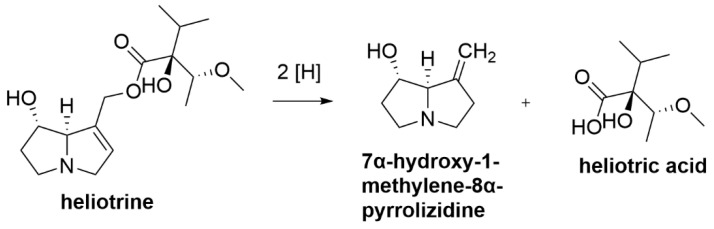
Proposed metabolic pathway of heliotrine degradation (adapted from [75]).

Resistance to PA poisoning was previously reported in sheep where the detoxification was hypothesised to occur in the rumen, suggesting that sheep rumen microorganisms have the ability to metabolise PAs and render them non-toxic [74,172]. Resistance against PA poisoning was reported to only occur in animals that were previously exposed to the toxin allowing rumen microbes to adapt to metabolise PAs. In confirmation, in vitro incubation of PAs that were commercially purchased or isolated from PA-containing seeds using ruminal contents from naïve sheep and cattle resulted in no PA degradation and the PAs remained stable in the incubation [173]. In contrast, sheep fed daily with 10 g/kg bw of dried *Heliotropium ovalifolium* (grey leaf heliotrope) did not show any poisoning symptoms [174] and no poisoning was reported in sheep fed daily with 105 mg/kg bw of *E. plantagineum* [175]. In vitro incubation of the PA heliotrine in rumen fluid from Australian Merino wethers, fed on green ryegrass-white clover diet, showed heliotrine degradation by a bacterium that was described to be Gram-negative, micrococcus, growing in pairs, short chains and clumps [75]. The bacterium was observed to reduce heliotrine (Figure 6) into 7α-hydroxy-1-methylene-8α-pyrrolizidine and heliotric acid in the presence of formate or molecular hydrogen as hydrogen donor [75]. Incubation of heliotrine in rumen fluid of Merino × Border Leicester ewes and wethers fed with dried *H. europaeum* resulted in the isolation of a bioactive strain which was named *Peptostreptococcus heliotrinreducens* [74]. This study was in agreement with Russell and Smith [75] and showed that formate and hydrogen were required for *P. heliotrinreducens* growth. *P. heliotrinreducens* was able to degrade other PAs including europine, lasiocarpine, supinine and heleurine to form 1-methylene derivatives similar to the metabolites observed by Russell and Smith [75] in modified media [74]. Both studies did not report any toxicity of the 1-methylene derivatives of PAs which suggest the possibility of successful PA detoxification by ovine rumen bacteria. This shows that PAs can be detoxified by reducing the 1,2 double-bond of the necine moiety. The presence of rumen bacteria in sheep that are able to detoxify PAs may be utilised for cross-inoculation to other ruminants thus conferring immunity to other ruminants when ingesting PAs.

A later study showed the synergistic relationship of ruminal microorganisms in metabolising *S. jacobaea* PAs. It was reported that a group of unidentified ruminal bacteria were responsible for PA metabolism while ruminal protozoa may have been involved in increasing the rate of degradation [172]. This could be due to the presence of protozoa and certain bacteria that could be responsible for the adsorption or adherence of PA-degrading bacteria to plant particles thus allowing the metabolising bacteria to utilise PAs [172]. 

Another in vitro incubation study compared the metabolism of monoester PA, diester PA and macrocyclic PA by either a mixed rumen bacteria culture (L4M2) obtained from rumen fluid of sheep that was fed with *S. jacobaea* as their diet or a commercially purchased *P. heliotrinreducens* (ATCC strain 29202) originally isolated from Australian sheep rumen fluid [76]. The study reported that *P. heliotrinreducens* was able to completely metabolise heliotrine (monoester PA) and lasiocarpine (diester PA) within 16 h of incubation but was unable to metabolise macrocyclic PAs throughout the 24 h incubation period [76]. The mixed L4M2 culture on the other hand was able to metabolise all three types of PAs and in shorter incubation times compared to *P. heliotrinreducens* [76]. All PAs were shown to be metabolised to their respective 1-methylene product and the L4M2 culture was found to be possibly able to further metabolise the 1-methylene PAs further [76]. Therefore, the L4M2 culture showed potential to be used as a probiotic against plants containing PAs. Isolation work of the L4M2 rumen microbial culture to identify rumen bacteria responsible for PA degradation resulted in the isolation of six bacterial isolates but these isolates were unable to degrade macrocyclic PAs and could not be identified using 16S rRNA gene sequencing [176]. Bacterial characterisation of enriched L4M2 culture using denaturing gradient gel electrophoresis and PCR cloning in a later study resulted in the classification of several genera of bacteria based on 16S rRNA gene sequences including *Anaerovibrio*, *Desulfovibrio*, *Megasphaera*, *Prevotella* and *Synergistes* [77].

### 2.4. Diterpenoids of Leafy Spurge

*Euphorbia esula* L. (leafy spurge) is a deep-rooted perennial, noxious weed growing in the rangelands and pastures in many areas of the northern Great Plains of North America and southern Canada since the early 1900s [177]. Leafy spurge has high nutritive value based on crude protein content with comparable values to other grazed pasture species such as alfalfa [178]. However, domesticated livestock, especially cattle, and wildlife tend to avoid grazing leafy spurge [33]. Since cattle are the predominant grazing livestock in North America, their aversion to leafy spurge is deemed to be one of the main reasons for the infestation of this introduced plant across two million acres of range and pasture lands in the United States alone [179]. Management strategies such as herbicides and intensive grazing were applied as a means to control leafy spurge infested lands but complete eradication of leafy spurge has been deemed to be economically unfeasible [180]. No poisoning of livestock from ingestion of leafy spurge in pastures and rangelands has been reported and this could be due to ruminants either avoiding grazing leafy spurge or having the ability to tolerate them. Not all grazing ruminants however were reported to have aversion to leafy spurge. Native sheep and goats were found to graze leafy spurge readily and domestic sheep were found to consume leafy spurge up to 50% of their daily dry matter intake [181]. Goats were observed to graze leafy spurge more willingly compared to sheep [182]. These observations suggest that goats and sheep may possess rumen microbial populations that are capable of metabolising leafy spurge secondary metabolites whilst the cattle rumen do not. The ability of sheep and goats to tolerate leafy spurge has been utilised as a biological control strategy to control leafy spurge invasion in pastures as this strategy is considered to be cost-effective and easy to implement compared to the use of herbicides [183]. 

**Figure 7:**
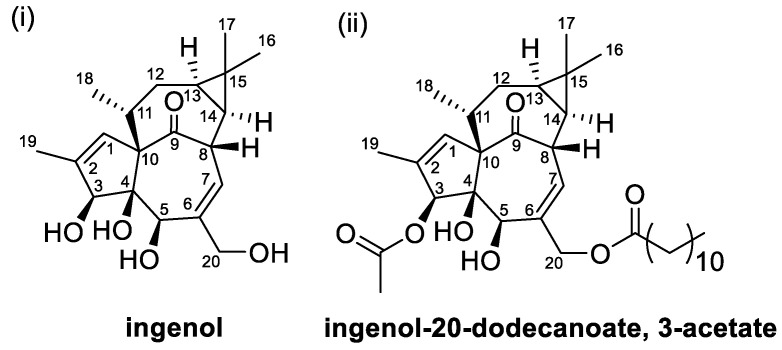
Chemical structures of (**i**) ingenol and (**ii**) ingenol-20-dodecanoate, 3-acetate.

Aversion to leafy spurge in cattle has been associated with condensed tannins and diterpenoids present in leafy spurge with these secondary metabolites being correlated with digestive upset in cattle when they ingest low doses of leafy spurge [184]. Ingenol (Figure 7i) and ingenol esters are some of the diterpenoids identified from extracts of leafy spurge which are found to be an irritant and a tumour promoter [34]. Both ingenol and its esters are suggested to induce toxicity due to their structural similarity to diacylglycerol and so mimic diacylglycerol thus activating protein kinase C [185]. The irreversible activation of protein kinase C has been documented to cause metabolic disruption leading to inflammatory response such as tissue damage, release of histamine causing irritation, unregulated cell growth and differentiation which could cause tumour formation [186].

Ingenol and ingenol esters are also reported to be partly responsible for the aversion developed in cattle to leafy spurge. Ingested ingenol was hypothesised to be absorbed into the blood from the gastrointestinal tract which then travels to the area postrema in the medulla oblongata in the brain leading to the development of conditioned aversion [187]. A feeding trial on cattle with feed treated with ingenol resulted in decreased feed intake by 83% on the second day while feed treated with ingenol-20-dodecanoate, 3-acetate (Figure 7ii) showed reduction of feed intake up to 89% on the second day [179]. Toxicity of isolated ingenol was found to be lower compared to its ester derivative of ingenol-20-dodecanoate, 3-acetate when tested on bovine lymphosarcoma cells [179]. The lower toxicity of ingenol compared to its ester also suggest that ingenol esters to elicit more aversion response than the parent ingenol. This could be due to ingenol esters being more lipophilic than ingenol thus they are more likely to be absorbed through the intestinal mucosa [188]. Leafy spurge extract incubated in pure cultures of common ruminal bacteria consisting of *Butyrivibrio fibrisolvens*, *Fibrobacter succinogens*, *Lachnospira multipara*, *Prevotella ruminicola*, *Selenomonas ruminantium* and *Streptococcus bovis* did not cause significant negative effect on their growth suggesting that the toxicity mechanism of leafy spurge extract did not involve the inhibition of feed digestion by rumen microbes [188].

Studies were done to determine the ability of rumen microorganisms to metabolise leafy spurge secondary metabolites. Sheep did not elicit aversion when fed with leafy spurge that was fermented in vitro in goat ruminal digesta but showed aversion to leafy spurge fermented in sheep ruminal digesta [78]. The study suggested that the aversive compounds present in leafy spurge were detoxified by rumen microorganism in goat rumen contents reflecting the difference in the ruminal microbiome of goats and sheep [78]. The study also confirmed previous observations that goats were able to graze leafy spurge more readily than sheep. Further studies to investigate the biotransformation of leafy spurge by rumen microorganisms and the identification of rumen microbes responsible for the degradation are required. In vitro digestibility of leafy spurge inoculated with rumen fluid of cattle or sheep that were either previously exposed or naïve to leafy spurge showed that the in vitro dry matter digestibility increased in rumen fluid previously exposed to leafy spurge while decrease in in vitro dry matter digestibility in naïve rumen fluid [180]. Another in vitro incubation study using rumen content of cattle and sheep fed with 2% dry matter/bw of leafy spurge reported improved dry matter digestibility compared to when using rumen content that was not previously exposed to leafy spurge [79]. The studies suggested that previous exposure to leafy spurge may alter the rumen microbial population to adapt to leafy spurge metabolites. However, this does not mean the adapted rumen digesta are able to detoxify toxic secondary metabolites of leafy spurge.

## 3. Rumen Microbial Detoxification of Cyanotoxins

Cyanobacteria (blue-green algae) can be commonly found in both fresh and sea waters. They play a key role in the ecosystem and biodiversity as primary producers, oxygen producers and in nitrogen fixation. Formation of algal blooms by cyanobacteria is caused by eutrophication which is an increasing environmental occurrence in the world. These blooms are considered to be potential health hazards as some cyanobacteria are able to produce cyanotoxins which are released into the water when the cells rupture or die. Every cyanobacteria species produces different cyanotoxins that can be classified according to the organs affected by toxicity. Generally, cyanotoxins vary in their toxicology. Chronic exposure of low doses of hepatotoxic cyanotoxins are associated with tumour growth, hepatocyte degeneration with necrosis, progressive fibrosis and mononuclear leukocyte infiltration [36,39]. Neurotoxic cyanotoxins are proposed to be a contributing factor for neurodegenerative diseases [37]. Cyanotoxins are also found to be protein inhibitors, causing DNA damage and genotoxicity and they induce oxidative stress [38,40,41]. Cyanotoxin exposure can occur directly or indirectly. Direct exposure of cyanotoxins mainly involves ingestion of contaminated water while indirect exposure includes consumption of animal or plant products contaminated with cyanotoxins. Cyanotoxins are reported to be able to bio-accumulate, causing their toxic effect to be magnified in food chains [189]. Reports of animal poisoning caused by cyanotoxins are well documented worldwide ranging from fish, domestic animals and livestock which include ruminants [35]. Livestock poisoning associated with cyanotoxins is a major concern as it causes economic losses and reduced animal performance. Sudden livestock death associated with cyanotoxin poisoning has been reported in Australia, United States and Canada [35].

**Figure 8:**
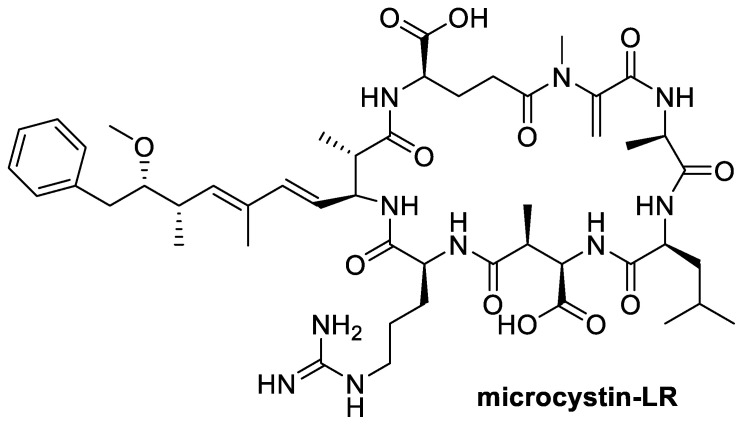
Chemical structure of microcystin-LR.

Microcystins are cyclic heptapeptides produced by cyanobacteria species of *Microcystis*, *Oscillatoria*, *Anabaena* and *Nostoc* and are known to be potent hepatotoxins [190] and tumour promoters [191]. Microcystin-LR (Figure 8) administered through intraperitoneal injection and oral administration to rats showed that rats given microcystin orally were less susceptible to the toxic effects compared to peritoneal administration, suggesting the role of detoxification by gut microorganisms or mechanisms that inhibited cyanotoxin absorption in the gastrointestinal tract [192]. Manubolu et al. [80] hypothesised that rumen microorganisms may have the ability to detoxify cyanotoxins and an in vitro incubation study was done using whole rumen contents from cattle that were not previously exposed to cyanotoxins. The rumen contents were incubated with cyanotoxins of microcystins (microcystin-LR, microcystin-RR and microcystin-YR) and nodularin for three hours and the degradation of the cyanotoxins were determined. Degradation of these cyanotoxins was observed with the degree of degradation determined to be dose and time dependent. Microcystins and nodularin showed higher rates of degradation of up to 36% by rumen fluid at low dose concentrations of 0.05 μg/mL while higher doses of cyanotoxins at 0.5 μg/mL and 5 μg/mL showed lower degradation rates and no degradation rate respectively [80]. Highest degradation rate was also observed in microcystin-YR at the first hour and rapidly decreased at the third hour [80]. The study showed the potential of rumen microbial flora to degrade cyanotoxins. However, no rumen microorganisms were isolated or identified and no metabolic pathway was determined from the incubation study.

## 4. Rumen Microbial Detoxification of Mycotoxins

Mycotoxins are secondary metabolites produced by fungi that can be toxic to both animals and humans [193]. Food and animal feed are highly susceptible to fungal growth and contamination in the field or during storage and the fungal species responsible determines the different mycotoxins which can be present. The Food and Agriculture Organisation (FAO) of the United Nations reported that 25% of the world’s food production is contaminated by at least one mycotoxin [194]. Commonly found mycotoxins include aflatoxin, fumonisins and trichothecenes which are large contributors to most agricultural mycotoxin poisoning concerns [195]. Clinical toxicological studies of high mycotoxin consumption showed various health issues ranging from acute mortality, slowed growth, gastrointestinal disorders, altered nutritional efficiency and reduced reproductive efficiency [196]. Monogastric animals are more susceptible to mycotoxins compared to ruminants as the rumen contains microorganisms which secrete enzymes that are capable of degrading the mycotoxins to less toxic or non-toxic metabolites [196,197,198]. However, not all mycotoxins can be detoxified by rumen microorganisms. Zearalenone which is a nonsteroidal estrogenic mycotoxin commonly associated with causing reproductive disorders in farm animals were found to form metabolites that are more toxic than the parent toxin [199,200].

### 4.1. Trichothecenes

Trichothecenes are a class of mycotoxins, produced mostly by a number of fungal species namely *Fusarium*, *Trichoderma*, *Cephalosporium*, *Myrothecium*, *Spicellum*, *Stachybotrys* and *Trichothecium,* which are harmful to human and animals’ health causing a wide range of acute and chronic symptoms [201]. 

**Figure 9:**
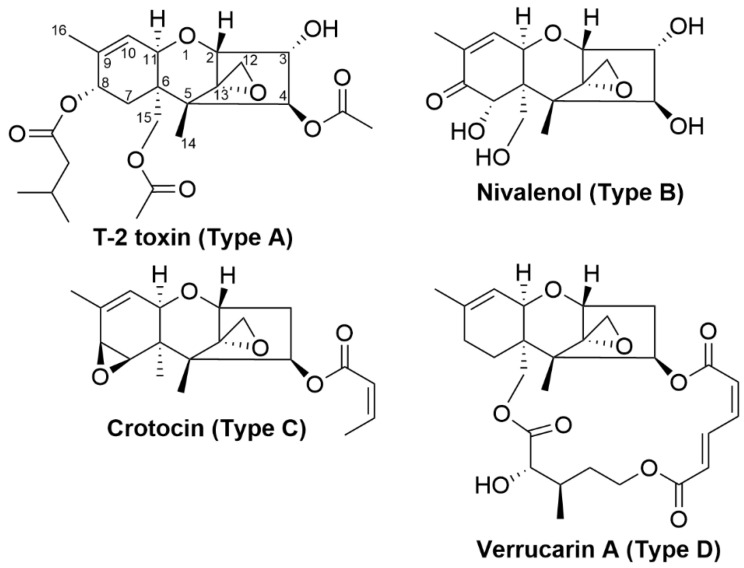
Trichothecene structures of Type A–D.

Trichothecenes share a common tricyclic 12,13-epoxytrichothec-9-ene core structure and are divided into four groups (types A–D) (Figure 9) depending on their producer fungi and substitution pattern on the core [201]. Both trichothecenes types A and B are chemically distinguished by the presence of an oxygen or carbonyl functional group at C_8_ position respectively. Type A trichothecenes such as neosolaniol which possess a hydroxyl group at C_8_ on its structure, T-2 toxin which has an ester function at C_8_ and diacetoxyscirpenol that has no oxygen substitution at C_8_ [201]. Nivalenol (NIV), deoxynivalenol (DON) and trichothecin are type B trichothecenes possessing a carbonyl group at C_8_ of their structure. Type C trichothecenes have a C_7_/C_8_ epoxide while type D trichothecenes have an additional ring that is linked via esters at the C_4_ and C_15_ hydroxyls [201]. This classification is commonly used to distinguish between trichothecenes but there are other structural features that are not accounted for in the system. 

Trichothecenes enter the host body through ingestion and are absorbed via the intergumentary and gastrointestinal systems as they can move passively across cell membranes. Trichothecenes are stable at neutral and acidic pH conditions making them stable and not hydrolysed in the stomach during digestion [201]. Toxicity of trichothecenes has been previously reviewed extensively [202,203]. Trichothecenes are toxic to all tested animal species but the sensitivity towards each trichothecene varies between organisms. Trichothecenes elicit toxic effects on animals which include immunosuppression, reduced growth rate, reproductive disorders, feed refusal and vomiting [42]. Early toxicity studies reported that trichothecenes inhibited eukaryotic protein synthesis by preventing the formation of peptide bond at the peptidyl transferase centre of the 60S ribosomal subunit affecting not only polypeptide chain initiation or elongation but also causing inhibition of polypeptide chain termination [43]. Trichothecenes are responsible for the inhibition of mitochondrial protein synthesis, inhibition of sulfhydryl enzymes and generation of free radicals causing harmful levels of oxidative stress [42,44]. Trichothecenes also inhibited cell growth and mitosis, with in vitro studies using both human cell lines and plants showing growth inhibition in every phase of the growth cycle [204].

**Figure 10:**
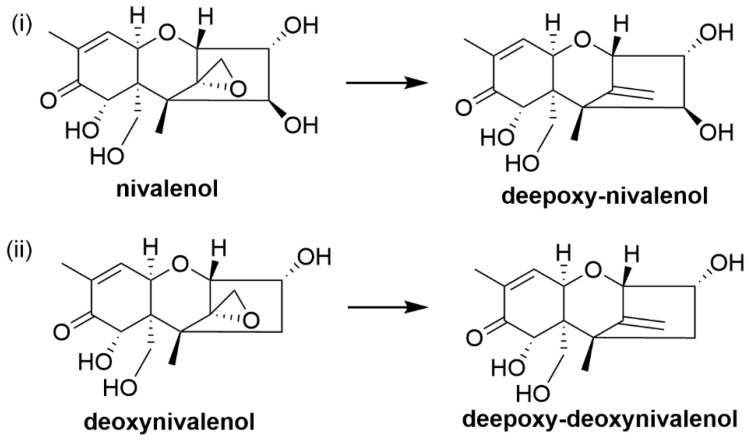
Proposed metabolic pathways of (**i**) NIV and (**ii**) DON degradation.

Only types A and B are commonly found in crops which are mainly produced by *Fusarium*. NIV and DON (Figure 10) are type B trichothecenes which are commonly found in cereals grown in temperate areas of America, Europe and Asia. The maximum allowable level of DON in food and feed set by the European Commission regulation (EU) 1881/2006, amended by the European Commission regulation (EU) 1126/2007 is 1250 µg/kg while the level of NIV was not set in the EU regulation [205]. NIV and DON are chemically stable due to their trichothecene skeleton and the epoxide ring is stable against nucleophilic attack. The epoxide group of trichothecenes is generally considered to be essential for its toxicity [206]. Detoxification of trichothecenes was previously observed when the oxygen in the epoxide group was removed to give a carbon-carbon double bond, resulting in non-toxic de-epoxy metabolites. De-epoxy T-2 toxin was observed to be 200 times less toxic than T-2 toxin in rat skin irritation assay [207]. Furthermore, de-epoxy T-2 toxin metabolites were 50 times less toxic compared to metabolites that had intact epoxides when tested in a brine shrimp LC_50_ test [208]. Cytotoxicity assays using a BrdU bioassay showed that de-epoxy NIV and de-epoxy DON (DOM-1) had higher IC_50_ values of 64.2 mM and 83 mM respectively, compared to NIV and DON, which had IC_50_ value of 1.19 mM and 1.5 mM respectively, suggesting the de-epoxides are less cytotoxic than their corresponding trichothecenes [209]. These studies suggested that the detoxification is a single step reaction where the epoxide is reduced thus reducing the toxicity effect of trichothecenes. 

The de-epoxide trichothecenes are suggested to be metabolites of intestinal or ruminal microbes as they were found in urine of rats and in urine and plasma of cattle [82,210,211]. Pig intestinal content and faeces microbes were found to transform trichothecenes to their respective de-epoxides [207,212,213]. The de-epoxidation step has been considered to occur in the intestines prior to absorption but the experimental evidence for this claim is weak as no de-epoxide metabolites were found in liver homogenates nor in in vitro incubation with pig intestinal content from duodenum or jejunum [212]. However, de-epoxide metabolites were found in in vitro incubations with intestinal content of pig caecum, rectum and colon [212]. 

It was previously reported that rumen microorganisms were capable of degrading NIV and DON into their respective de-epoxide metabolites (Figure 10). In vitro rumen fermentation of DON at concentrations equivalent to 5 ppm and 10 ppm in feed, showed about 89% of DON was reduced within 48 h of incubation [81]. The study also reported that the amount of trichothecenes in the feed can affect the rate of transformation such that when DON was incubated at concentrations equivalent to 50 ppm and 100 ppm in feed, only 49% and 38% of DON was transformed respectively by rumen microbes [81]. In vitro incubation of 2 ppm NIV and DON in rumen fluid collected from Swedish Red and White cows for 48 h resulted in 82% of NIV and DON converted into de-epoxy NIV and DOM-1 respectively [83]. Results also suggest that de-epoxidation reaction is relatively slow as only 35% of DON was found to be de-epoxidised after six hours of incubation [83]. An in vivo trial in which lactating Holstein cows were fed naturally DON-contaminated corn showed DON to be metabolised to DOM-1 and the metabolite was found to be excreted in urine, plasma and milk [82]. Other DON metabolites were also identified from studies involving orally administered DON into sheep where glucuronide conjugated DON, glucuronide conjugated DOM-1 and sulfate conjugated DON were detected in urine and bile [84]. Anaerobic in vitro incubation of 1000 ppm DON in rumen fluid collected from cannulated cattle showed 35% of DON was de-epoxidised after 96 h [85].

**Figure 11:**
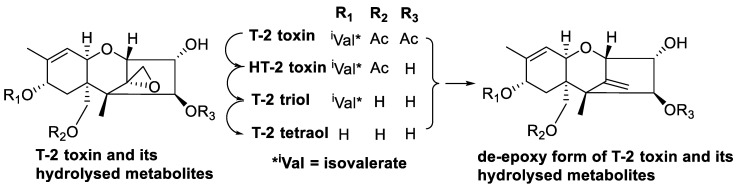
Proposed T-2 toxin and T-2 metabolite degradation pathway (adapted from [86]).

**Figure 12:**
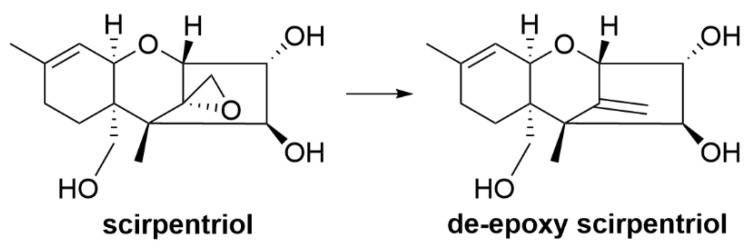
Proposed metabolic pathway of scirpentriol degradation (adapted from [86]).

A study using in vitro incubation of 100 ppm DON in cattle rumen fluid showed biotransformation of DON into DOM-1 and resulted in the isolation of a bioactive microorganism, identified as an anaerobic Gram-positive bacterium [214]. Further study of the bacterium indicated a new species within the genus *Eubacterium,* designated *Eubacterium* strain BBSH 797 [215]. The isolated strain was further developed into a commercial product for trichothecene detoxification in animal feed designed for poultry and swine diets under the brand name Mycofix^®^. A modified in vitro test model with pig intestine inoculated with *Eubacterium* strain BBSH 797 and incubated with DON showed formation of DOM-1 [88]. Toxicity test showed that DOM-1 was 500 times less toxic than DON [88]. In vitro incubation of BBSH 797 with type A trichothecenes, T-2 toxin and scirpentriol showed that T-2 toxins undergo both hydrolysis of the acetyl group and de-epoxidation into non-toxic T-2 metabolites (Figure 11) while scirpentriol was de-epoxidised into non-toxic de-epoxy scirpentriol (Figure 12) [86].

### 4.2. Aflatoxin B_1_

Aflatoxins are naturally occurring mycotoxins and potent carcinogens produced mainly by fungi of the *Aspergillus* family. They are known to be main contaminants of a variety of tropical and subtropical food and feed stuffs. Since the discovery of aflatoxins, many studies were done on their negative effects in laboratory animals and livestock which include effects on animal performance, toxin metabolism and carryover of toxin residues to animal products [216]. Aflatoxins are difuranocoumarin derivatives consisting of a few groups namely B_1_, B_2_, G_1_, G_2_, M_1_ and M_2._ Aflatoxin B_1_ (AFB_1_) is considered to be the most common and most toxic aflatoxin [217]. The maximum allowable level of AFB_1_ in food and feed set by the European Commission regulation (EU) 1881/2006, amended by the European Commission regulation (EU) 165/2010 is 2 µg/kg [218]. AFB_1_ is known to be hepatotoxic and carcinogenic [219,220], and also causes other negative effects that can be either directly or indirectly associated with the toxicity such as immunosuppression, reduced feed utilisation, reduced animal productivity and growth rate. Susceptibility to AFB_1_ varies for every animal species with different acute toxic manifestations. AFB_1_ hepatocarcinogenesis was reported in various animal species where fish and poultry were found to be extremely sensitive to AFB_1_ while mice and rats can tolerate higher concentrations of AFB_1_ before showing hepatic tumours [219,221]. Cattle consuming a sufficient dose of aflatoxins can have negative affects to their health, performance and reproduction [45]. Cattle affected by aflatoxicosis are reported to suffer from adverse health effects which include weight loss, liver damage, decreased milk yield and reduced feed utilisation efficiency [46].

**Figure 13:**
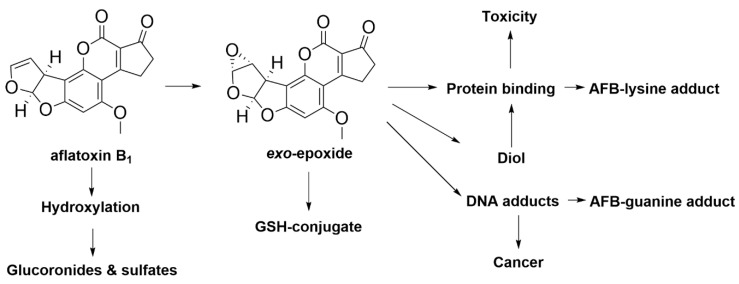
Metabolism and toxicity mode of action of AFB_1_ [222].

Mechanisms of AFB_1_ absorption and metabolism were previously studied and reported in both animals and humans [217,222,223]. AFB_1_ metabolism mainly happens in the liver but AFB_1_ can be also metabolised in the kidneys as residues can be detected from urine. AFB_1_ can be metabolised by a range of cytochrome P450s which include CYP1A2, CYP3A4 and CYP2A6 from the liver and other tissues [217]. Cytochrome P450 enzymes (CYP450) in the liver metabolise AFB_1_ into aflatoxin M_1_ (AFM_1_), aflatoxin P_1_ (AFP_1_), aflatoxin Q_1_ (AFQ_1_) and aflatoxicol. The toxicity mechanism of AFB_1_ is described as below (Figure 13). AFB_1_ can be metabolised to form epoxides AFB_1_-8,9-*exo*-epoxide and AFB_1_-8,9-*endo*-epoxide in the endoplasmic reticulum. AFB_1_-8,9-*exo*-epoxide is highly unstable and reacts more readily with DNA to form AFB_1_-N^7^-guanine adducts by intercalation of epoxide between base pairs which can induce cell mutations causing cancer compared to AFB_1_-8,9-*endo*-epoxide. Similar to AFB_1_, metabolite AFM_1_ can be activated to form AFM_1_-8,9-epoxide and bind to DNA forming AFM_1_-N^7^-guanine adducts. The epoxides are also able to bind with other macromolecules such as protein to induce toxicity and be further metabolised into AFB_1_-N^7^-lysine adducts by the formation of covalent bonds between the epoxide and serum albumin. Both guanine and lysine adducts of AFB_1_ and AFM_1_ are found to be excreted in urine. AFP_1_, AFQ_1_ and aflatoxicol are excreted in urine or in feces in the form of glucuronyl conjugates from bile. The epoxides can be also conjugated to form GSH-conjugates which can be further detoxified by glutathione S-transferases. AFB_1_-8,9-epoxides can be further hydrolysed to form AFB_1_-8,9-dihydrodiol which can also bind to proteins to form AFB_1_-lysine adducts. Alternatively, AFB_1_ can be also hydroxylated via monooxygenases and also form glucuronide and sulfate conjugates.

With the wide-spread effects of AFB_1_ on animal health and the risk of mycotoxin contamination in food products for human consumption, it is not surprising that there are many studies being done on ways to control and detoxify mycotoxins. Current strategies used to detoxify mycotoxins include physical, chemical and biological methods. Physical and chemical detoxification methods such as thermal inactivation and addition of chemical absorbents in feed were found to be time consuming, costly or only partially effective and not able to be upscaled for real-world application [224]. Adsorbent feed additives such as activated charcoal and hydrated sodium aluminium silicates at low feed inclusion rates were not effective in binding aflatoxins while these adsorbents, if used at high feed inclusion rates, cause binding of essential nutrients [225]. Therefore, use of enzymes or microorganisms has emerged as an alternative strategy to detoxify aflatoxins in animals as they are considered to be an effective and safer detoxification method.

AFB_1_ detoxification by rumen microorganisms was among the earliest mycotoxins reported to be degraded in the rumen. Early studies on aflatoxin excretion of sheep and cattle in England that were orally dosed with pure mixed aflatoxins B_1_, G_1_ and B_2_ reported that AFB_1_ levels in urine and faeces of cattle were higher compared to sheep. The metabolite AFM_1_ which was excreted by cattle was only 4.09% of the AFB_1_ administered from the oral dose, while 3.55% of AFB_1_ was unmetabolised [226]. The result suggested that remaining AFB_1_ had undergone degradation to non-toxic metabolites. The authors argued that the higher levels of AFB_1_ in urine and faeces of cattle than in sheep suggested sheep rumen microbes may have greater AFB_1_ detoxification activity compared to cattle. Incubation of AFB_1_ in vitro in rumen fluid of domesticated Holstein steers and native goats in Korea reported AFB_1_ degradation of about 14% and 25% in steers and goats respectively [227]. This study did not report the identification of any rumen microorganisms nor detect any AFB_1_ metabolites.

There are also studies reporting the isolation of bacterial strains capable of detoxifying AFB_1_. Although the isolated bacteria were not of rumen origin, these bacteria could be potential probiotics for ruminants which can be applied to AFB_1_ contaminated feed. Isolated *Bacillus* strains from Thai fermented soybean product incubated in vitro with pure AFB_1_ showed the *Bacillus* strains were able to degrade between 50% and 70% of AFB_1_ [228]. Degradation was observed to occur within the first 5–6 days of incubation followed by AFB_1_ concentrations remaining constant until the end of the incubation time [228]. A gut bacterium isolated from fish gut was found to be able to degrade 81.5%, 60% and 80.7% of AFB_1_, AFM_1_ and AFG_1_ respectively in vitro [229]. The microbe responsible for the degradation was identified to be a strain of *Bacillus subtilis* [229]. Broilers that were fed a basal diet containing AFB_1_ and a combination of the bacteria *Lactobacillus casei*, *Bacillus subtilis* and *Pichia anomala* did not suffer negative effects of AFB_1_ on chicken’s production performance [230], and the authors suggested AFB_1_ was successfully detoxified by the combination of these probiotics.

**Figure 14:**
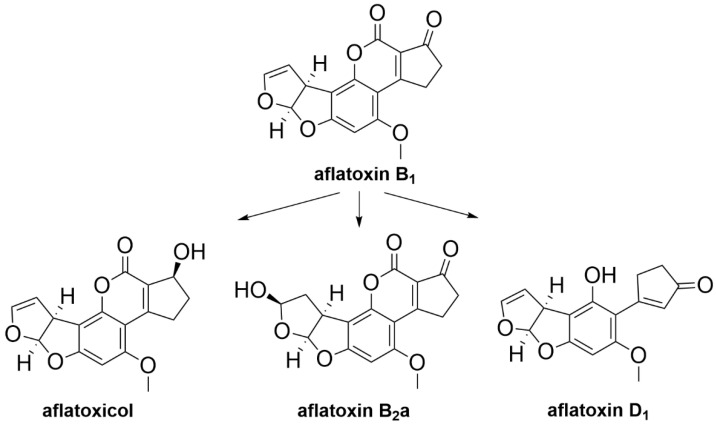
Proposed metabolites of AFB_1_ degradation (adapted from [87]).

Degradation metabolites of AFB_1_ have been previously identified. AFB_1_ metabolites aflatoxicol, aflatoxin B_2_a (AFB_2_a) and aflatoxin D_1_ (AFD_1_) are degradation products produced by *Streptococcus* spp. and *Lactobacillus* spp. which are facultative anaerobes that can be present in the rumen (Figure 14) [87]. In vitro toxicity studies reported that these metabolites were found to be less toxic than AFB_1_. Aflatoxicol was found to be 18 times less toxic compared to AFB_1_ but was able to form adducts with DNA. AFB_2_a is 200 times less toxic than AFB_1_ and is considered to be relatively non-toxic. AFD_1_ was found to be non-toxic based on in vitro toxicity studies using Hela cells [87,231,232].

### 4.3. Ochratoxin A

Ochratoxin A (OTA) is a mycotoxin produced by *Aspergillus* and *Penicillium* fungi and OTA is found to be a natural contaminant in foodstuffs of plant origin in tropical, subtropical and temperate regions [47]. In areas with cooler climates, OTA tends to be produced by *Penicillium* fungi while *Aspergillus* fungi tend to produce OTA in warmer climates [233]. The maximum allowable level of AFB_1_ in food and feed set by the European Commission regulation (EU) 1881/2006, amended by the European Commission regulation (EU) 594/2012 is 3 µg/kg [234]. OTA exposure in animals is shown to be mainly nephrotoxic showing other pathological responses which are hepatotoxic, teratogenic and carcinogenic [47,48]. Furthermore, OTA toxicity in animals can also cause potential indirect human exposure to OTA through animal derived food where trace OTA was detected in meat, milk, dairy products and other animal derived foodstuffs [235,236,237]. OTA is considered to be the cause for the Balkan endemic nephropathy which is a human kidney disease caused by tumours in the urinary tract leading to irreversible kidney damage [238]. In vitro and in vivo studies suggest that OTA exposure to animal/human cells, resulted in the overproduction of free radicals resulting in oxidative stress [51,52,53]. Studies showed that OTA is responsible for lipid peroxidation whereby OTA-Fe^3+^ complex was reduced to OTA-Fe^2+^ complex in the presence of NADPH-CYP450 reductase initiating the formation of free radicals [54,239]. OTA could induce DNA strand breaks and induce the production of oxidative chromatin and DNA damage in vitro, inhibiting the growth of umbilical cord matrix mesenchymal stem cells [50,240]. The Phe moiety of OTA could compete with Phe for binding to Phe-tRNA synthetase thus inhibiting peptide elongation [241]. 

The bioavailability of OTA varies depending on the food matrix. In vitro digestion model studies suggest 30–100% of OTA to be bioavailable while some OTA that was not absorbed in the upper gastrointestinal tract could travel down to the colon and be absorbed into the body [242,243]. OTA generally has a longer half-life in blood than in tissues due to the higher binding affinity of OTA to blood proteins [244]. Animal studies also showed that OTA was found to be distributed mostly in the kidneys follow by liver, muscle and fat consistent with OTA as nephrotoxic [47]. OTA metabolites have been characterised in different species based on in vitro and in vivo studies. The major metabolic pathway of OTA includes the toxin being biotransformed through hydrolysis, hydroxylation, opening of the lactone ring and conjugation. OTA was shown to undergo hydroxylation by both phase I and phase II CYP450 enzymes forming hydroxyl metabolites, (4*R*)-4-hydroxyochratoxin A ((4*R*)-OH-OTA), (4*S*)-4-hydroxyochratoxin A ((4*S*)-OH-OTA), 5′-hydroxyochratoxin A, 7′-hydroxyochratoxin A and 10-hydroxyochratoxin A [47,245,246]. Oxidation of OTA by CYP1A1/1A2 and CYP3A1/3A2 by rat liver microsomes also produced (4*R*)- and (4*S*)-OH-OTA [247]. CYP450 enzymes oxidise OTA to produce OTA-quinone which could be further be conjugated with GSH or reduced to form OTA-hydroquinone [248]. OTA can be biotransformed to ochratoxin B which is a dechlorinated form of OTA by renal microsomes [49]. OTA metabolites were found to be less toxic than OTA however their toxic effect is still strong enough to induce OTA toxicity [47,249].

OTA residues can be found in animal tissues which include the muscles, liver, kidney and serum [250]. OTA feed contamination causes nephropathy in pigs with observed progressive interstitial fibrosis and regressive tubular changes with thickening of the basement membrane in the porcine kidney [251]. Chickens fed with OTA were observed to have swollen kidneys, yellowish livers, hyperplasia of the binary epithelium and hypertrophy of renal proximal tubular epithelial cells [252]. Ruminants on the other hand are rarely reported to be acutely susceptible to OTA while the harmful effects of OTA are usually due to the prolonged ingestion of the toxin instead [250]. However, OTA was found to be absorbed relatively quickly into the bloodstream of ruminants which could be of health concern for ruminants due to accumulation of OTA in the bloodstream [250]. Despite ruminants being reported as more resistant to OTA poisoning, there are still some reports on ochratoxicosis in ruminants. Ewes fed with OTA through intravenous infusion died within 24 h [55]. Goats that were fed with daily oral doses of OTA at 3 mg/kg bw were reported to have died within five days of feeding [56].

**Figure 15:**
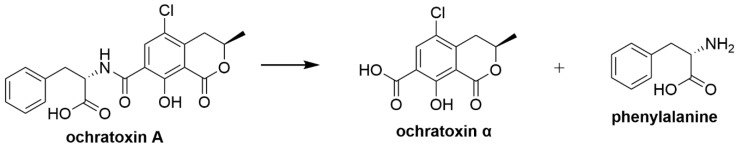
Proposed detoxification pathway of OTA in the rumen.

The chemical structure of OTA consists of a dihydroisocoumarin named ochratoxin α (OTα) which is linked through a C_9_ carboxyl group (attached at C_7_), to L-α-phenylalanine by an amide bond [47]. Non-ruminants are found to be more susceptible to OTA while ruminants are relatively resistant to OTA due to their ability to detoxify OTA [253]. OTA absorbs readily to the gastrointestinal tract of monogastrics with little or no degradation, while OTA in ruminants undergoes microbial degradation in the rumen before absorption into the bloodstream [48]. The most common microbial degradation of OTA is the hydrolysis of the amide bond of OTA releasing the non-toxic OTα and phenylalanine (Figure 15). Anaerobic microorganisms in the large intestine and cecum of monogastrics [254] and in the rumen are found to be responsible for the degradation. Ruminants are considered to be less susceptible to OTA compared to monogastric animals as the hydrolysis of OTα in rumen can occur before absorption and is considered the principal OTA detoxification method in ruminants [255]. OTA that was administered into calves through a stomach tube resulted in death within 24 h supporting the claim that the rumen is responsible for OTA degradation [56]. Furthermore, pre-ruminant calves were also observed to be susceptible to OTA with the calves dying within the first 24 h of dosing with OTA [255].

Proteolytic enzymes such as carboxypeptidase A, chymotrypsin, protease A and pancreatin are such enzymes reported to show OTA degradation activity, usually by cleaving amide bonds at the carboxy-terminal end of a peptide [48]. It has been generally accepted that rumen protozoa are associated with OTA degradation instead of rumen bacteria due to the presence of high proteolytic activity which allows the hydrolysis of the amide bond of OTA. The role of protozoa as OTA degraders was reported in an in vitro OTA incubation study, with rumen contents from cattle and sheep where OTA transformation to OTα was observed in the total rumen fluid content and the protozoal fraction [89,90]. In vitro OTA degradation was observed in inoculum contents from three pre-gastric forestomach compartments, the rumen, reticulum and omasum while no degradation was observed from inoculation into the fourth compartment, the abomasum or true stomach [91]. In vitro OTA incubation in rumen fluid from Korean native goats showed OTA degradation in whole rumen fluid and the bacterial fraction containing bacteria and protozoa [92]. Further gene amplification of carboxypeptidase A from goat rumen fluid showed the gene was expressed from *Bacillus lichenformis* and *Bacillus* spp. populations in the rumen [92]. Another in vitro study also reported on OTA degradation by *B. lichenformis* although the isolate was not of rumen origin [228]. In vitro degradation studies using rumen fluid incubated with OTA, followed by enrichment and culture dilution, isolated a rumen bacterium capable of cleaving OTA into OTα and phenylalanine while partial 16S rRNA gene sequence analysis of the bacterium showed that it is closely related to *Lactobacillus vitulinus* [88]. Different feed types are found to influence OTA degradability in ruminal fluid. A study reported that OTA degradation was higher in ruminal contents of sheep fed with hay compared to sheep that was fed with cereal [256]. Overall, the studies suggest that high forage diets favour microbial populations in the rumen capable of OTA degradation.

### 4.4. Fumonisins

**Figure 16:**
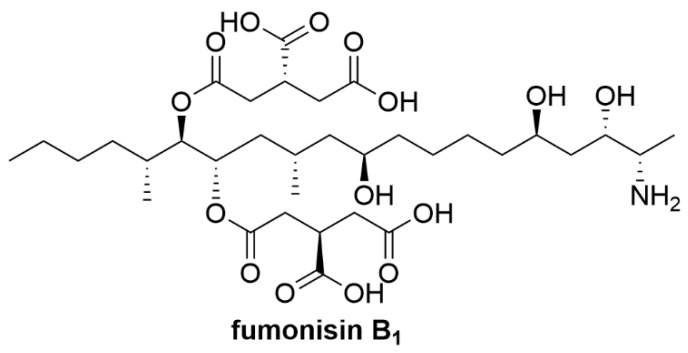
Chemical structure of fumonisin B_1_.

Fumonisins are a group of mycotoxins produced commonly by *Fusarium verticillioides* and *F. proliferatum* which contaminate cereal grains. High levels of fumonisins in contaminated crops are found to commonly occur in locations with warmer climates especially during drought seasons when crop plants undergo heat stress [257]. Fumonisins are classified in four groups which are A, B, C and P however only fumonisins of group A (A_1_ and A_2_) and group B (B_1_, B_2_ and B_3_) are commonly discussed and studied [258]. Fumonisin B_1_ (Figure 16) is the most commonly studied fumonisin and has been linked to leukoencephalomalacia in horses, pulmonary oedema in swine and hepatocarcinoma in rats [259]. The maximum sum allowable levels of fumonisin B_1_ and fumonisin B_2_ in food and feed set by the European Commission regulation (EU) 1881/2006 is 4000 µg/kg [260]. Epidemiological studies linked fumonisin B_1_ to oesophageal cancer in humans [261]. Fumonisins are structurally similar to sphingoid bases of sphinganine and sphingosine by possessing an unsubstituted primary amino group at C_2_ which competitively inhibits ceramide synthase thus disrupting the synthesis of ceramide and the metabolism of spingolipids [262]. This leads to the accumulation of sphinganine and sphingosine in the liver and kidney cells causing metabolic effects such as mitochondrial apoptosis, impaired sphingolipids involved in synthesis and transport, degeneration of sphingolipid-rich tissues and increasing oxidative stress and lipid peroxidation [258].

Toxicological and pathological effects of fumonisins in animals are well documented [258,259]. Pharmacokinetic studies on rodents and primates showed that ingested fumonisins were absorbed in the gastrointestinal tract, rapidly distributed in blood and accumulated mostly in liver and kidneys [259]. Fumonisin toxicity was found to be dependent on sex, genus and dose-response differences in animals. Female mice were found to be more susceptible to nephrotoxicity compared to male mice [263] while rabbits were more sensitive to the toxic effects of fumonisin B_1_ compared to rats [259]. Horses are the most susceptible to fumonisins with leukoencephalomalacia being a disease of the nervous system causing necrosis, softening, cavitation and yellow discolouration in the grey and white matter of the cerebral hemispheres [264]. Clinical symptoms in affected horses include muscle weakness, blindness, heart weakness, coma and death in severe cases [264]. Onset of toxicity occurs when horses consume 10 ppm fumonisin contaminated feed with symptoms showing within 7–180 days [265]. Consumption of 92 ppm fumonisin B_1_ in contaminated feed by pigs, caused death within 48 h of ingestion [258]. Poultry are less sensitive to fumonisins where those with diets containing 100–400 mg/kg of fumonisins B_1_ showed liver necrosis, biliary hyperplasia and heart damage [266].

Ruminants are reported to be tolerant towards fumonisins although there are reports that ruminants can develop toxicosis when fed with high concentrations of fumonisins. Fumonisin contaminated feed up to 148 ppm caused mild liver lesions in feeder calves and lymphocyte blastogenesis [57]. Holstein milk-fed calves fed with 1 mg/kg bw of fumonisin B_1_ for seven days showed hepatic and renal damage but cardiovascular function remained unaffected [58]. Oral feeding of fumonisins from *F. verticilloides* cultures to lambs and goat kids also resulted in similar mild hepatic and renal damage [59,60]. Ruminant resistance to fumonisins at low concentrations suggested the possibility that rumen microorganisms may be able to metabolise fumonisins. Reports on fumonisin B_1_ degradation by rumen contents are inconsistent. Studies of incubation of fumonisin B_1_ in rumen fluid of cattle for 72 h only showed up to 18% degradation with no metabolites detected [93]. In vitro incubation of fumonisin B_1_ at varying concentrations (50 ppm and 100 ppm) for 72 h resulted in only 10% degradation irrespective of fumonisin B_1_ concentration [94]. Another study reported that fumonisins incubated in cattle rumen fluid at concentration of 100 ppm and 200 ppm showed no effect on volatile fatty acid (VFA) production but decreased branched chain VFAs and ammonia concentrations were observed, indicating the possibility of a shift in rumen bacterial populations in the presence of fumonisins [267]. No metabolites of fumonisin B_1_ were identified from these studies and the fumonisin concentration used in the studies were high enough to induce toxicosis. 

## 5. Conclusions

This review has highlighted the potential use of rumen microbial metabolism of natural toxins to improve ruminant health and welfare. Rumen microorganisms have been shown to have the ability to metabolise and detoxify a wide range of plant toxins and mycotoxins. Feeding a toxin to ruminants in low doses for a prolonged period of time has also demonstrated the potential to stimulate a shift in rumen microbial populations to utilise a toxin. It has been also shown that some toxins can be rendered non-toxic by a simple hydrolysis step using a rumen bacterium while other toxin metabolites may require multiple metabolic reactions involving various rumen microorganisms for detoxification. Inoculation of rumen contents of ruminants that are resistant to a certain toxin into naïve ruminants has been shown to transfer toxin resistance. Further work is however required to understand the effect of rumen microbial population shifts on the metabolic activity in the rumen. There is also the need to identify rumen microorganisms responsible for detoxification and to investigate the metabolic pathways of detoxification. Toxin metabolites also need to be studied to determine their toxicity and chemical structure to further understand the mechanism of action of the toxin. A complete understanding of the detoxification by candidate rumen microorganisms would lead to the development of safe and effective probiotics for ruminants. The number and diversity of toxin candidates presented in this review highlights the potential to extend this approach to other toxins for which the capacity for rumen microbial detoxification has not yet been explored. The review also shows the commercial potential of rumen inoculum derived probiotics as safe and viable alternatives to improve ruminant health and production while being cost-effective and timesaving compared to other conventional toxin management strategies such as chemical detoxification and pasture management.

## Figures and Tables

**Table 1:** Summary of toxins and their effects on ruminants.

Toxin Name	Toxin Source	Effects on Ruminants
**Mimosine**	*Leucaena leucocephala*	Hair loss, goitre, poor cattle live-weight gain [14] and hypothyroidism [15]
**4-*N*-acetyl-2,4-diaminobutyric acid**	*Acacia angustissima*	Head pressing, grinding of teeth, foaming at the mouth and jerking of the body [16]
**β-*N*-oxalyl-l-α,β-diaminopropionic acid**	Grass pea (*Lathyrus sativus*)	Lathyrism [17], induces oxidative stress and excitotoxicity resulting in motor neuron degeneration [18]
**Indospicine**	*Indigofera* spp. (particularly *I. linnaei* and *I. spicata*)	Hepatotoxic, teratogenic, loss in body weight, embryo-lethal effects [19,20,21,22] and reproductive losses [20,23,24]
**Fluoroacetate**	Naturally in over 40 plant species. Also used as pesticide (Compound 1080)	Death [25,26], acidosis, hypocalcaemia and heart failure [27]
**Pyrrolizidine alkaloids**	About 3% of all flowering plants, including *Heliotropium*, *Senecio*, *Crotalaria*, *Echium*, and *Cynoglossum* species	Loss of appetite, diarrhoea and depression [28] Death [29,30,31] and megalocytosis [32]
**Ingenol and ingenol esters**	Leafy spurge (*Euphorbia esula* L.)	Aversion to plant [33], irritant and tumour promoter [34]
**Microcystins and nodularin**	Cyanobacteria	Sudden death, reduced animal performance [35] and potential to be carcinogenic, hepatotoxic, immunotoxic, neurotoxic and genotoxic [36,37,38,39,40,41]
**Trichothecenes (Nivalenol, deoxynivalenol and T-2 toxin)**	Fungi including *Fusarium*, *Trichoderma*, *Cephalosporium*, *Myrothecium*, *Spicellum*, *Stachybotrys* and *Trichothecium*	Immunosuppression, reduced growth rate, reproductive disorders, feed refusal, vomiting [42], eukaryotic protein synthesis inhibition [43] and generation of free radicals [44]
**Aflatoxin B_1_**	*Aspergillus* fungi	Reduced animal health, performance, reproduction [45], weight loss, liver damage, decreased milk yield and reduced feed utilisation efficiency [46]
**Ochratoxin A**	*Aspergillus* and *Penicillium* fungi	Nephrotoxic, hepatotoxic, teratogenic, carcinogenic [47,48,49,50], formation of free radicals [51,52,53,54] and fatal poisoning [55,56]
**Fumonisins**	*Fusarium verticillioides* and *Fusarium proliferatum* fungi	Liver, kidney damage [57,58,59,60] and lymphocyte blastogenesis [57]

**Table 2:** Rumen microorganisms and their role in toxin degradation to non-toxic metabolites.

Toxin	Identified Rumen Microorganisms	Role in Toxin Degradation
**Mimosine**	*Synergistes jonesii*	Degrades toxic mimosine metabolites, 3,4-dihydroxypyridine and 2,3-dihydroxypyridine into unidentified non-toxic metabolites [15,61,62,63]
**4-*N*-acetyl-2,4-diaminobutyric acid**	Bacteria not identified	Hydrolyses 4-*N*-acetyl-2,4-diaminobutyric acid to diaminobutyric acid and diaminopropane followed by further degradation into non-toxic metabolites [64]
**Diaminopropionic acid**	*Firmicutes* strain LPLR3; *Klebsiella* strain LPSR1	Degrade diaminopropionic acid to further non-toxic metabolites [64]
**β** **-*N*-oxalyl-** **l** **-** **α** **,** **β** **-diaminopropionic acid**	*Megasphaera elsdenii*; *Clostridium bifermentans*	Degradation pathway and metabolites not identified [65,66]
**Indospicine**	Bacteria not identified	Hydrolyses indospicine to 2-aminopimelamic acid and 2-aminopimelic acid followed by further metabolism to hypothesised non-toxic metabolites [67]
**Fluoroacetate**	*Synergistes*, *Pigmentiphaga, Ancylobacter, Pyramidobacter* spp.; *Butyrivibro fibrisolvens* genetically modified with dehalogenase gene from *Moraxella* sp. strain B	Degrade toxic fluoroacetate into non-toxic fluoride and acetate [68,69,70,71,72,73]
**Pyrrolizidine alkaloids**	*Peptostreptococcus heliotrinreducens*; L4M2 mixed rumen bacterial culture from sheep rumen, containing bacterial species *Anaerovibrio*, *Desulfovibrio*, *Megasphaera*, *Prevotella* and *Synergistes*	Reduce pyrrolizidine alkaloid, heliotrine into non-toxic 7α-hydroxy-1-methylene-8α-pyrrolizidine and heliotric acid [74,75,76,77]
**Ingenol and ingenol esters**	Bacteria not identified	Degradation pathway and metabolites not identified [78,79]
**Microcystins and nodularin**	Bacteria not identified	Toxin degradation observed but degradation pathway was not identified [80]
**Nivalenol and deoxynivalenol**	*Eubacterium* strain BBSH 797	Nivalenol and deoxynivalenol degraded into their less-toxic de-epoxide metabolites [81,82,83,84,85]
**T-2 toxin and scirpentriol**	*Eubacterium* strain BBSH 797	T-2 toxin and scirpentriol degraded into their less-toxic de-epoxide metabolites [86]
**Aflatoxin B_1_**	*Streptococcus* sp. and *Lactobacillus* sp. which may be present in the rumen	Degradation of aflatoxin B_1_ into less toxic aflatoxicol, less toxic aflatoxin B_2_a and non-toxic aflatoxin D_1_ [87]
**Ochratoxin A**	Rumen protozoa; *Bacillus lichenformis*; *Lactobacillus vitulinus*	Hydrolysis of ochratoxin A into non-toxic ochratoxin α and phenylalanine [48,88,89,90,91,92]
**Fumonisins**	Bacteria not identified	Degradation pathway and metabolites not identified [93,94]
